# Terrible Case of Arsenical Poisoning

**Published:** 1879-11

**Authors:** 


### EDITORIAL, ETC.

*Terrible Case of Arsenical Poison.*—One of the most unfortunate and tragical terminations of the possibly careless use of arsenious acid in a tooth, occurred a few days ago in Brooklyn, N. Y., full accounts of which were published in the daily papers. It sounds almost like an impossible story that even the careless use of this substance could cause the results thus reported.

"Mr. George Arthur Gardner, nephew by marriage of Prescott, the historian, died in Brooklyn on the 27th ult., in great agony, after two weeks of indescribable suffering. It is said by his attending physician that his death was caused by arsenical poison placed by a dentist in one of his teeth for the purpose of killing an aching nerve. The certificate of death, which was filed by Dr. Samuel S. Guy, states that the cause of death was "gangrene of mouth and face, arising from treatment of a tooth." Mr. Gardner was nearly fifty years old, a married man, and by occupation a civil engineer. His illness began two weeks before his death, his tooth first having been treated by a Boston dentist, and afterwards by a Brooklyn dentist. Mr. A. C. Lewis, a friend of the deceased, who was with him during his last illness, said: "No man ever died such a terrible death as Gardner died. When he was dead every connection between his head and body, except the spine, had been eaten through and completely severed by the action of the poison. Every time a blood-vessel was severed there would be a new hemorrhage. A few days before he died one of these hemorrhages suddenly occurred, and the blood spouted up into the attendant's face. The sloughing of the decayed parts was so great that four incisions had to be made into his neck to keep his throat clear. Through these incisions we had continually to draw out the sloughed parts, and they came in long strips like soft gum, and had to be broken off. I suppose if he could have done so he would have killed himself rather than remain so offensive. He was dying while alive all the time."

A correspondent of a Boston paper, who interviewed Dr. Waters, reports him as stating that Mr. Gardner came to his office in Boston on Tuesday afternoon, September 9th, and complained of a severe toothache. Mr. Gardner said that a dentist had examined the tooth and had put in an application, which, however, had had but little effect, as the aching had been stopped but temporarily. Dr. Waters said that he examined Mr. Gardner's tooth and found that the aching member was the first molar in the right lower jaw. Mr. Gardner requested Dr. Waters to stop the pain and plug the tooth, and the latter did so. He found the tooth very badly decayed. He washed out the cavity with a solution of carbolic acid to remove the decayed matter and food which had settled in the tooth, applied nitrate of silver to the nerve and then plugged the tooth with gutta-percha to keep out the air and food. After the operation he advised Mr. Gardner to see another dentist in a few days, after his return to Brooklyn, N. Y., in order that the tooth might be permanently filled, and to call again and see him (Dr. Waters) on his next visit to Boston. "Mr. Gardner," Dr. Waters said, "was naturally very robust, and was the picture of health, but he did not appear to be as well as usual when he visited me." Dr. Waters, said Mr. Gardner complained of not feeling well when he left his office." Dr. Waters did not hear anything of him again until a week afterwards, when he was informed that he had been prostrated with what was stated to be arsenical poison, used in filling the tooth. Dr. Waters said that he had also been informed that Mr. Gardner had been treated by Dr. Marvin, a dentist of Brooklyn, N. Y. He heard nothing further until Saturday evening, September 27th, when Mr. Joseph B. Gardner, a brother of the deceased, called on him and stated that George had died. Dr. Waters said that he used no arsenic, nor any preparation containing arsenic in treating Mr. Gardner's tooth. He never used arsenic in his profession, but knew that some dentists used the poison. He said that he should not be surprised if the dentist who had performed the first operation on the tooth, as stated by Mr. Gardner, when he visited Dr. Waters in Boston, had used arsenic, and that Mr. Gardner's complaint of poor health was due probably to the poison which at that time had been absorbed.

